# The relationship between sleep quality and psychological resilience of college students: the examination of insomnia as a mediator and attachment as a moderator

**DOI:** 10.3389/fpsyg.2025.1640656

**Published:** 2025-09-12

**Authors:** Yi Zhang, Juanjuan Li, Jianyu Dong, Lingling Shi, Ning Zhang

**Affiliations:** ^1^Department of Neuropsychiatry and Clinical Psychology, Beijing Tiantan Hospital, Capital Medical University, Beijing, China; ^2^School of Marxism, Capital Normal University, Beijing, China; ^3^Beijing Tiantan Hospital, Capital Medical University, Beijing, China

**Keywords:** college students, sleep quality, psychological resilience, insomnia, attachment security

## Abstract

**Background:**

Sleep quality received widespread attention as a significant factor influencing psychological resilience. Existing research has inadequately addressed the relationship and the mechanism between sleep quality and the psychological resilience of college students.

**Objective:**

This study aims to explore the relationship between sleep quality and psychological resilience among college students, while examining how insomnia and attachment security mediate this association. This study may provide advice for promoting the sleep quality and psychological resilience of college students.

**Methods:**

A total of 585 college students from multiple universities in Beijing were selected through convenience sampling via questionnaire survey. Data was collected using students’ self reported sleep quality with the Pittsburgh Sleep Quality Index (PSQI), insomnia symptoms with the Insomnia Severity Index (ISI), attachment security with Adult Attachment Scale (AAS) and psychological resilience with Psychological Resilience Scale (PSR). Robust linear regression model and structural equation model were employed to analyze the relationship between sleep quality and psychological resilience among college students.

**Results:**

University students in Beijing show a certain degree of sleep problems (mean PSQI = 8.27, SD = 3.56), with 43.59% having experienced insomnia. Their psychological resilience is relatively good (mean = 3.48, SD = 1.81). There is a positive correlation between sleep quality and insomnia index among college students (*β* = 1.36, *p* < 0.001); poorer sleep quality is associated with lower psychological resilience (*β* = −0.02, *p* < 0.05), higher insomnia index is associated with lower resilience (*β* = −0.01, *p* < 0.1), and students with insecure attachment exhibit lower psychological resilience compared to those with secure attachment (*β* = −0.50, *p* < 0.001). Of the total effect of sleep quality on psychological resilience, 39.24% is mediated through insomnia, while the remaining 60.76% is a direct effect. Adult secure attachment can effectively moderate the sleep–insomnia pathway. This research may help to understand the relationship among sleep quality, attachment, and psychological resilience.

## 1 Introduction

Sleep plays an important role in both physical and mental health (Scott et al., 2021). College students are facing increasing academic and employment pressures, and their sleep problems are becoming increasingly severe, study showed that 40–60% of American college students had sleep problems and a third had a shortened sleep duration (Becker et al., 2018), 20.3 and 23.6% of Chinese students have sleep complaints and suffering from insomnia (Li et al., 2018). Lack of sleep can lead to a range of problems, such as physical discomfort, depressive moods, and other mental health issues (Alvaro et al., 2013). Compared to primary and secondary school students, working adults, and the elderly, college students are generally in their best physical condition, and less attention has been paid to their sleep problems.

Psychological resilience refers the ability to adjust to stressful circumstances and persevere in the face of adversity (Masten et al., 1990). Studies have found that high levels of resilience can improve mental health and psychological well-being (Dray et al., 2017). Psychological resilience has attracted attention from the positive psychology field due to its important role. Existing studies often focus on the resilience of specific populations, such as military personnel, patients, and people with depression (Klatt et al., 2019), while relatively little attention has been given to the resilience of college students. Sleep, as an important factor affecting mental health, also has a certain influence on psychological resilience. Previous research often considers sleep and psychological resilience as joint factors explaining mental health, stress perception and emotions (Tomioka et al., 2024; Poon et al., 2024). Alternatively, psychological resilience is used as a mediating variable to explain the effects of sleep on various mental health outcomes (Chatburn et al., 2013). Some studies also explore the impact of sleep quality on psychological resilience in specific populations. For example, some research has pointed out that dance students with poor self-reported sleep quality showed a higher risk of low resilience because having good sleep quality may help to decrease stress response (Arbinaga, 2018). Sleep disturbance is a modifiable threat to mental and physical resilience, which can be mitigated by intervention strategies that promote consolidated, restorative, and sufficient sleep (Germain and Dretsch, 2016). Sleep problems and psychological resilience were strongly correlated, sleep problems were found to be predictive of resiliency scores. Resiliency significantly mediated the relationship between increased sleep problems and both overall internalizing and externalizing behavior problems, and specifically, measures of depression and anxiety (Chatburn et al., 2013). Existing research has paid insufficient attention to the relationship between sleep and psychological resilience among college students, and the underlying mechanisms linking the two remain underexplored. This study aims to focus on the college student population and examine how sleep quality affects their psychological resilience.

### 1.1 Psychological resilience of college students

Psychological resilience can be defined as an ability for individuals to positively cope with adversity and encourages positive adjustment and development when facing challenging circumstances (Rutter, 2006). Psychological resilience can act as a protective factor for mental health by reducing the negative psychological impact of stressors (Mealer et al., 2012). The importance of psychological resilience for college students comes from the multiple challenges and stressors they are facing throughout their academic journey. Resilience plays a crucial role in helping students to regulate emotions and to overcome test anxiety, resilience-training intervention may be developed to support students encountering anxiety during the exam (Liu et al., 2021). Resilience can also help students to prevent the onset of anxiety symptoms via improve sleep quality (Cai et al., 2023). Research shows that psychological resilience is the mediating variable of the influence of learning stress on learning burnout (Gong et al., 2023). College managers and teachers should attach importance to the development of college students’ psychological resilience and shape their good psychological quality so that they can gain a foothold in the society (Sherblom et al., 2022). Affect-regulation frame of psychological resilience has proposed four strategies to enhance psychological resilience including situation change, attentional deployment, cognitive change, and response modulation (Troy et al., 2023). Researches focusing on the cognitive mechanism to resilience like physical activity interventions and their cognitive benefits, directly relating to resilience are becoming popular (Dhahbi et al., 2025). Sleep quality, insomnia and adult attachment are factors on cognitive, which is important for psychological resilience.

### 1.2 Sleep quality and psychological resilience

Research shows that good sleep quality is essential to psychological well-being and poor sleep is one of the key correlates of poor mental health (Scott et al., 2021). For college students, chronic sleep deprivation may bring lower academic performance and an increased risk of burnout (Naderi et al., 2021) and if the sleep problems persist for a long time, they may suffer emotional problems such as anxiety and depression (Marvaldi et al., 2021) and even increasing the risk of suicidal ideation (Guo, 2021). Environmental factors such as blue light exposure are important factors to affect the sleep quality of college students (Souissi et al., 2025). Good sleep quality may enhance resilience and decrease stress, so sleep is a modifiable behavior (Lo Martire et al., 2024). A meta-analysis on 63 articles demonstrated a clear positive relationship between sleep (quality and quantity) and psychological resilience. It is showed that longer sleep duration and better sleep quality are related to higher levels of resilience with the strongest effect observed for sleep quality (Arora et al., 2022).

### 1.3 Insomnia and psychological resilience

The Insomnia Severity Index (ISI) is one of the commonly used scales for measuring sleeping quality. It assesses an individual’s sleeping condition by evaluating insomnia disorders. Insomnia often manifests as an insomnia disorder, which is one of the most common sleep disorders. It is characterized by frequent and persistent difficulty in falling asleep or maintaining sleep, leading to inadequate sleep satisfaction (American Academy of Sleep Medicine, 2014). Insomnia significantly impairs daytime alertness and has been identified as both a precursor and a maintaining factor for various physiological diseases and mental disorders (Baglioni et al., 2014). Research suggests that insomnia may serve as the underlying mechanism linking sleeping quality and psychological resilience (Lo Martire et al., 2024), meaning that sleeping quality influences psychological resilience through insomnia.

### 1.4 Attachment and psychological resilience

Attachment, which initially used to analyze children’s psychological health issues, refers to the strong and enduring emotional bond formed between an infant and their primary caregiver (Bowlby, 1985) and later extended to adult intimate relationships (Hazan, 1987). As a psychological state that helps individuals coping with adversity and stress, psychological resilience is also influenced by attachment styles. Research has shown that psychological resilience is affected by attachment styles, and mentalization can influence college students’ psychological resilience through the mediating role of attachment styles, such as dismissive attachment, secure attachment, and fearful attachment (Wang et al., 2023). Both attachment anxiety and attachment avoidance may significantly reduce individuals’ well-being by increasing their psychological inflexibility and decreasing their resilience and mindfulness (Calvo et al., 2022). Research shows that attachment may effect mindfulness which may positively predict resilience, it is possible to facilitate attachment security through cultivating trait mindfulness, and in this way, resilience could be enhanced (Yang and Oka, 2022).

The 3P model of insomnia includes importance of predisposing, precipitating, and perpetuating factors (Spielman et al., 1987). Stress is the most common initiating factor and patients with insomnia show increased cognitive and psychological response to stress according to the hyperarousal model (Riemann et al., 2015). Attachment is important factors for understanding the relationship between stress and response to stress. Securely attached individuals have less anxiety and depression symptoms compared to insecurely attached individuals, and they think that they receive more social support and less stressed from their environment (Priel and Shamai, 1995), perceived stress and psychological symptoms are more common in insecurely attached individuals (McCarthy et al., 2006). Studies show that insecure attachment may have an influence on insomnia perpetuating factors by negatively influencing pre-sleep state arousal and emotion regulation (Palagini et al., 2018).

Based on existing research, this study aims to further explore the underlying mechanisms of the relationship between college students’ sleeping quality and psychological resilience. It aims to enhance the research framework while also helping researchers develop more targeted interventions and guidance strategies. By doing so, this study seeks to support college students in coping with various challenges more proactively and improving their psychological adaptability. Based on the above analysis, this study proposes the following hypotheses:

*H1*: There is a correlation between sleep quality and psychological resilience among college students. The better the sleep quality, the higher the level of psychological resilience.

*H2*: The more severe the insomnia experienced by college students, the lower their level of psychological resilience.

*H3*: College students with secure attachment exhibit higher levels of psychological resilience.

*H4*: Sleep quality affects psychological resilience through the mediation of insomnia. That is, poorer sleep quality leads to more severe insomnia, which in turn leads to lower psychological resilience.

*H5*: Secure attachment moderates the relationship between sleep quality and insomnia. College students with secure attachment are less likely to experience insomnia.

## 2 Data and methods

### 2.1 Data and samples

Data was collected through online survey with a convenience sampling method. The online survey was conducted via Wenjuanxing among students from multiple universities, including Beijing Foreign Studies University, Capital Medical University, Capital Normal University, and Beijing University of Agriculture and so on. A total of 642 questionnaires were collected. The questionnaires were distributed to students through school counselors and teachers, and collected through voluntary completion by the students. The ethical review of this research has been approval from the ethics committee of the first hospital of Jilin university (No.23 K133-001). After reviewing the response time and logical consistency, 57 invalid questionnaires were excluded, resulting in 585 valid responses, with an effective recovery rate of 91.1%. Research findings specify that a minimum sample size of 494 is necessary to maintain a sampling error of ±4.5% with 95% confidence (De Vaus and De Vaus, 1986). Although this study employs non-probability sampling, with 585 sample size, this study remains a meaningful reference. Similar research has utilized convenience sampling with sample sizes around 500 for analytical purposes. As a non-probability sampling study, the conclusions are confined only to the surveyed sample. However, given the commonalities among university students, the findings can offer reference significance for other collegiate populations.

### 2.2 Research measurement

#### 2.2.1 Pittsburgh Sleep Quality Index (PSQI)

PSQI is a self-reported sleeping assessment scale developed by Buysse et al. (1989). PSQI index is used to measure the sleep quality of the students. The scale consists of 18 items and covers seven dimensions, namely sleep quality, sleep latency, sleep duration, sleep efficiency, sleep disturbances, sleep medication, and daytime dysfunction. The PSQI adopts a 4-point Likert scale (0–3 points), with a total score > 7 indicating the poor sleep quality issue. Higher scores (score >7) indicate worse sleep quality. In this study, the calculated Cronbach’s alpha coefficient for PSQI was 0.8209, suggesting good internal consistency.

#### 2.2.2 Insomnia Severity Index (ISI)

ISI is a self-assessment tool developed by Morin (1993) to evaluate individuals’ perception of insomnia symptoms over the past 2 weeks. Both ISI and PSQI are commonly used tools to assess sleeping quality. Compared to PSQI, ISI specifically evaluates the severity of insomnia. The scale consists of seven items measuring various aspects of insomnia, including sleep distress, falling asleep difficulty, and maintaining sleep difficulty. It uses a 5-point Likert scale (0–4 points), with a total score ranging from 0 to 28. Lower scores indicate better sleep quality with no symptoms of insomnia, while higher scores indicate more severe insomnia. In this study, the calculated Cronbach’s alpha coefficient for ISI was 0.9236, indicating good internal consistency.

#### 2.2.3 Psychological Resilience Scale (PSR)

PSR score is used in this study to measure the psychological resilience of college students. PSR used in this study is a revised version developed by Hu and Gan (2008). The scale consists of 27 items (e.g., “My life has a clear goal”) and assesses five dimensions: goal orientation, emotional regulation, positive cognition, family support, and social support. It uses a 5-point Likert scale (1–5 points), where 1 represents “completely disagree” and 5 represents “completely agree.” The scale includes 12 reverse-scored items. Higher scores indicate better psychological resilience. In this study, the calculated Cronbach’s alpha coefficient for the scale was 0.9158, indicating good internal consistency.

#### 2.2.4 Adult Attachment Scale

AAS scale is used to measure the attachment style of college students. AAS used in this study is the 1996 revised version by Collins (1996). The scale consists of 18 items measuring adult attachment, divided into three subscales: closeness, dependence, and anxiety. Each subscale is assessed using six items. The scale adopts a 5-point Likert scale (1–5 points), where 1 represents “completely disagree” and 5 represents “completely agree.” It includes six reverse-scored items. The closeness score is the average score of item 1, 6, 12 and reverse-score of items 8, 13, 17; the independent score is the average score of item 5, 14, 16, and reverse-score of item 2, 7, 18; the anxiety score is the average score of item 3, 4, 9, 10, 11, 15. As Chinese students often tend to choose the midpoint score (neutral choice), it is difficult for several students to be classified. Considering that both independent and closeness types represent secure attachment patterns, this study adjust the three subscales (closeness, dependence, and anxiety) into two attachment types: Secure Attachment, in which Individuals scoring >3 on closeness and dependence, scoring <3 on anxiety. Those with secure attachment value relationships, experience both intimacy and autonomy, and hold a positive view of both themselves and others in interpersonal relationships. Insecure Attachment, in which individuals who either view themselves positively but others negatively or hold relatively negative views of both themselves and others. They tend to exhibit anxiety or emotional instability in relationships.

### 2.3 Statistical methods

Data were collected and organized using Excel, and StataMP17 was used for data analysis. Descriptive statistical analysis was conducted on the demographic characteristics of college students. One-way ANOVA and independent sample t-tests were used to analyze differences between groups. The Harman single-factor method was applied to test for common method bias. Given the non-significant heteroscedasticity test (Breusch-Pagan χ^2^(1) = 0.00, *p* = 0.95), standard OLS estimation procedures with robust standard errors were applied to examine the relationships between sleeping quality, insomnia index, adult attachment styles, and psychological resilience. Additionally, a structural equation model (SEM) was developed to analyze the mediating effect of insomnia on the relationship between sleeping quality and psychological resilience, as well as the moderating effect of adult attachment styles on the relationship between sleep quality and psychological resilience.

## 3 Results analysis

### 3.1 Distribution of research subjects

A total of 585 valid questionnaires were collected in this survey. As a convenience sampling study, the representativeness of the sample remains compromised—an acknowledged limitation of this research that will be elaborated in subsequent sections. Among the collected samples, 431 were from female students (73.68%) and 154 from male students (26.32%). The students were from multiple universities, including Beijing University of Agriculture and Beijing Foreign Studies University. By university classification, 400 students were from Double First-Class universities, making up 68.38%, while 184 students were from non-Double First-Class universities, accounting for 31.45%. One student did not specify their university. By age group, 516 students were aged 18–22, making up 88.2%, while 49 students were 22 years or older, accounting for 8.4%. Twenty students did not specify their age.

### 3.2 Common method bias test

As all variables in this study were collected through an anonymous online questionnaire completed by college students, the data were collected through convenience sampling. The Harman single-factor test was applied to test for common method bias (Fan et al., 2011). The variance explained by the largest factor among those with eigenvalues greater than 1 was 23.02%, which is well below the commonly accepted threshold of 40%. This indicates that there is no significant common method bias in this study. This phenomenon may be associated with the design of the questionnaire and the data collection methodology employed.

### 3.3 Descriptive statistics and correlation coefficients of variables

The result of the descriptive statistics of variables is showed in Table 1 as follows. Overall, college students exhibit some sleeping problems, with an average sleep quality score of 8.27 (a score greater than 7 indicates sleep problems, indicating that the surveyed university students experience significant sleep-related issues.). There is considerable individual variation in sleep quality, with a variance of 3.56. Students also experience some degree of insomnia, though not severe, with an insomnia index of 7.47. There is substantial variation in insomnia levels among students, with a variance of 6.34. In total, students demonstrate middle grade psychological resilience, with little variation among individuals. The average psychological resilience score is 3.48, with a variance of 1.81.

**Table 1:** Descriptive statistics of variables.

Continuous variables				
Variables	Means	Variance	Minimum	Maximum
Sleep quality	8.27	3.56	0	20
Insomnia index	7.47	6.34	0	28
Psychological reliance	3.48	0.54	1.81	4.85

Categorical variables			
Variables	Ratio	Variables	Ratio
Adult attachment (Secure)	45.47%	Drowsiness: ≥3 times per week	32.82%
Adult attachment (Insecure)	54.53%	Lack of energy: none	18.46%
No insomnia	56.41%	Lack of energy: occasionally	46.67%
Mild insomnia	28.55%	Lack of energy: sometimes	19.83%
Moderate insomnia	12.31%	Lack of energy: frequently	15.04%
Severe insomnia	2.74%	Extremely low psychological resilience	0.17%
Sleep: more than 7 h	28.21%	Low psychological resilience	15.04%
Sleep: 6–7 h	55.38%	Moderate psychological resilience	67.01%
Sleep: 5–6 h	14.70%	High psychological resilience	17.78%
Sleep: less than 5 h	1.71%	Bedtime: 18:00–21:00	1.03%
Drowsiness: none	13.68%	Bedtime: 21:00–23:00	13.16%
Drowsiness: less than once per week	16.41%	Bedtime: 23:00–24:00	40.51%
Drowsiness: 1–2 times per week	37.09%	Bedtime: after 24:00	45.30%

To further explore the details of students’ sleep quality, a detailed analysis of bedtime, sleep duration, and other sleep-related factors was conducted. The results revealed that 71.79% of students sleep less than 7 h per night, while 16.41% sleep less than 6 h, indicating significant sleep problems. Students tend to go to bed relatively late, with 85.81% going to bed after 11 PM, and as many as 45.3% going to bed after midnight. Additionally, daytime functional impairment due to poor sleep quality is also a concerning issue. 69.9% of students reported feeling drowsy at least once per week, while 32.82% experienced drowsiness more than three times per week. Furthermore, 15.04% of students frequently felt a lack of energy while performing tasks, and 34.87% reported often feeling fatigued.

To assess the insomnia much more precisely, a classification of insomnia severity was conducted by dividing ISI scores into four categories:0–7, No insomnia; 8–14, Mild insomnia; 15–21, Moderate insomnia; 22–28, Severe insomnia. Observing the distribution across these categories, the findings indicate that 43.59% of students experience insomnia, with 15.01% suffering from moderate to severe insomnia. These results highlight the importance of addressing students’ sleep habits and insomnia issues.

To further explore students’ psychological resilience levels, resilience scores were categorized into four levels: 1.0–1.99, Extremely low psychological resilience; 2.0–2.99, Low psychological resilience; 3.0–3.99, Moderate psychological resilience; 4.0–5.0, High psychological resilience. The distribution analysis revealed that most students had moderate or higher psychological resilience. However, 15.21% of students exhibited low psychological resilience, while 17.78% demonstrated high psychological resilience. A detailed analysis of students’ adult attachment styles showed that 45.47% of students had a secure attachment style, while 54.53% exhibited an insecure attachment style. These findings suggest that students’ perception and management of interpersonal relationships still require further improvement.

A detailed analysis of students’ adult attachment styles showed that 45.47% of students had a secure attachment style, while 54.53% exhibited an insecure attachment style. These findings suggest that students’ perception and management of interpersonal relationships still require further improvement.

Considering the types of variables, Pearson correlation, Point-Biserial Pearson correlation, and Spearman rank correlation were used to examine the relationships between sleep quality, insomnia index, adult attachment, and psychological resilience. The results are shown in Table 2.

**Table 2:** Correlation coefficients of variables.

Variables	Sleep quality	Insomnia index	Adult attachment	Psychological attachment
Sleep quality	1.00			
Insomnia index	0.77***	1.00		
Adult attachment	0.21***	0.23***	1.00	
Psychological attachment	−0.34***	−0.33***	−0.53***	1.00

***p < 0.001, **p < 0.05, *p < 0.01.

Sleep quality and insomnia index showed a significant positive correlation (Spearman rank correlation coefficient = 0.77, *p* < 0.001), indicating that poorer sleep quality is associated with more severe insomnia.

Sleep quality and adult attachment were significantly positively correlated (Point-Biserial Pearson correlation coefficient = 0.21, *p* < 0.001), suggesting that individuals with poorer sleep quality tend to exhibit insecure attachment styles.

Sleep quality and psychological resilience had a significant negative correlation (Pearson correlation coefficient = −0.34, *p* < 0.001), indicating that worse sleep quality is associated with lower psychological resilience.

Insomnia index and adult attachment were significantly positively correlated (Spearman rank correlation coefficient = 0.23, *p* < 0.001), suggesting that individuals with severe insomnia are more likely to exhibit insecure attachment characteristics.

Insomnia index and psychological resilience showed a significant negative correlation (Spearman rank correlation coefficient = −0.33, *p* < 0.001), indicating that more severe insomnia is associated with lower psychological resilience.

Adult attachment and psychological resilience had a significant negative correlation (Point-Biserial Pearson correlation coefficient = −0.53, *p* < 0.001), meaning that individuals with insecure attachment characteristics tend to have lower psychological resilience.

### 3.4 Analysis of differences in various scales

Independent sample t-tests were conducted with gender, age group, and school category as independent variables and sleep quality and psychological resilience as dependent variables. For insomnia index, non-parametric Wilcoxon rank-sum tests (equivalent to the Mann–Whitney U test) were performed using gender, age group, and school category as independent variables. To examine differences in attachment styles, Chi-square tests were conducted with gender, age group, and school category as independent variables. The results, presented in Table 3, indicate:

Significant gender differences in adult attachment styles.
Significant differences in psychological resilience among students from different school categories.
Significant differences in sleep quality and insomnia index among different age groups.

**Table 3:** Statistical differences in sleeping quality, insomnia index, adult attachment, and psychological resilience across factors.

Variables	Groups	Sleeping quality x_±s	Insomnia index (rank sum)	Adult attachment(secure/insecure) frequency		Psychological reliance x_±s
Gender	Male	8.12 ± 0.30	46,125	59	95	3.42 ± 0.04
	Female	8.32 ± 0.17	125,280	207	224	3.51 ± 0.03
	Pearson chi^2^(1)			4.32		
	P score	0.56	0.58	0.04		0.07
School type	Double-first-class	8.11 ± 0.17	115,130	188	212	3.53 ± 0.03
	None double-first-class	8.58 ± 0.29	55,690	78	106	3.39 ± 0.04
	Pearson chi^2^(1)			1.08		
	P score	0.17	0.32	0.30		0.01
Age group	22 years below	8.17 ± 0.16	143,386	230	286	3.49 ± 0.02
	22 years above	9.73 ± 0.52	16,509	23	26	3.41 ± 0.07
	Pearson chi^2^(1)			0.10		
	P score	0.00	0.02	0.75		0.32

### 3.5 Relationship among sleep quality, adult attachment, and psychological resilience

A robust linear regression model was conducted to explore the relationship between sleeping quality and insomnia index (computed case was 564). The results indicate that, after controlling gender, age group, and school category, sleep quality significantly affected the insomnia index, that is, lower sleep quality was associated with a higher insomnia index (*β* = 1.36, *p* < 0.001, model R^2^ = 0.59).

Furthermore, robust linear regression models were conducted to examine the effects of sleep quality, insomnia index, and insecure attachment on psychological resilience. The results are presented in Table 4. After controlling gender, school category, and age group, sleeping quality, insomnia index and adult attachment were sequentially added to the regression models, and the R^2^ value increased from 0.13 to 0.33. After controlling gender, age group, and school category, the results showed that:

Sleep quality (*β* = −0.02, *p* < 0.05) had a significant negative effect on psychological resilience, that means lower sleep quality was associated with lower psychological resilience.
Insomnia index (*β* = −0.01, *p* < 0.1) had a marginally significant negative effect on psychological resilience, that means higher insomnia index was linked to lower psychological resilience.
Adult attachment (*β* = −0.50, *p* < 0.001) had a strong negative effect on psychological resilience, that means students with insecure attachment had significantly lower psychological resilience compared to those with secure attachment.

**Table 4:** Regression model with psychological resilience as the dependent variable.

Variables	Model 1	Model 2	Model 3
Sleep quality	−0.050***	−0.031***	−0.023**
Insomnia index		−0.014**	−0.009*
Insecure attachment			−0.497***
Female	0.09*	0.08	0.034
None-double-first class	−0.09*	−0.09*	−0.09**
22 years above	0.01	0.02	−0.02
R^2^	0.12	0.14	0.34
ΔR^2^		0.01	0.20
Cohen’s f^2^ (*Δ*)		0.01	0.30
Overall Cohen’s f^2^	0.14	0.16	0.51
Prob > F	0.00	0.00	0.00
VIF	1.51	1.59	1.51

***p < 0.001, **p < 0.05, *p < 0.1.

### 3.6 Mediating and moderating effects of insomnia and attachment

To further explore the mechanism underlying the sleep quality–insomnia–psychological resilience relationship, a structural equation model (SEM) was constructed to analyze the mediating effect of insomnia index on the relationship between sleep quality and psychological resilience. The bootstrap resampling method (based on a non-normally distributed sample distribution) was applied, with 5,000 bootstrap samples drawn to estimate the 95% confidence interval of the mediating effect. Gender, age group, and school classification were included as control variables, and the mediating role of insomnia index in the relationship between sleep quality and psychological resilience was analyzed. The results are presented in Table 5 computed cases was 564, the root mean square error of approximation (RMSEA) was 0.000 (with both bounds of the 90% confidence interval also at 0.000 and pclose = 1.000, indicating negligible approximation error. The comparative fit index (CFI) and Tucker–Lewis index (TLI) both reached 1.000, well above the ideal threshold of 0.95. The standardized root mean square residual (SRMR) was 0.000, far below the recommended maximum of 0.08, suggesting a high degree of consistency between predicted and observed values. The Bentler–Raykov squared multiple correlation indicated that the model accounted for approximately 60.6% of the total variance, reflecting strong explanatory power. Overall, the model demonstrated excellent fit quality and effectively captured the structural relationships among the variables). The findings revealed the following:

Sleep quality significantly negatively predicted psychological resilience (*p* = 0.001), indicating that poorer sleep quality is associated with lower psychological resilience.
Insomnia index significantly negatively predicted psychological resilience (*p* = 0.001), suggesting that higher insomnia index is linked to lower psychological resilience.
Sleep quality significantly positively predicted insomnia index (*p* < 0.001), indicating that poorer sleep quality is likely to lead to a higher insomnia index.
Sleep quality indirectly influenced psychological resilience through the insomnia index, and this indirect effect was significant (*p* = 0.01). This suggests that insomnia index plays a partial mediating role in the relationship between sleeping quality and psychological resilience.

**Table 5:** Decomposition of indirect and direct effect of sleeping quality on psychological resilience.

Path	Effect	Boot (SE)	Boot Z score	P score	Boot 95% CI	Effects ratio (%)
Direct effect	−0.03	0.01	−3.31	0.001	[−0.05,-0.01]	60.76
Indirect effect	−0.02	0.01	−2.57	0.011	[0.03,-0.00]	39.24
Total effect	−0.05	0.01	−7.90	0.000	[0.06,-0.04]	100

The total effect of sleep quality on psychological resilience was significant (*p* < 0.001), meaning that sleeping quality affects psychological resilience both directly and indirectly through insomnia.

The indirect effect accounted for 39.24%, indicating that 39.24% of the impact of sleeping quality on psychological resilience is mediated by insomnia index, while the remaining 60.76% is a direct effect.

To assess the robustness of the mediation effect, we conducted a sensitivity analysis by estimating the indirect effect under different model specifications (without covariates, controlling only for gender, and controlling for all covariates). In all three specifications, the indirect effect of psqi → isi → prs remained statistically significant (*p* < 0.01), positive, and of similar magnitude. These results suggest that the mediation effect is robust to alternative model specifications.

To further explore the mechanisms underlying the relationships among sleep quality, adult attachment, insomnia index, and psychological resilience, a multiple-group structural equation model (MSEM) was used to examine whether adult attachment style moderates the mediation pathway of sleep quality → insomnia index → psychological resilience (case computed was 564, The model’s fit indices all met or exceeded internationally recognized standards. RMSEA = 0.000, with both bounds of its 90% confidence interval also equal to 0.000, indicating negligible approximation error and excellent model fit. CFI = 1.000 and TLI = 1.000, both well above the recommended threshold of ≥0.95, demonstrating outstanding goodness of fit. SRMR = 0.000, significantly lower than the recommended cutoff of 0.08, indicating minimal residuals and a high degree of consistency between observed and predicted values. The coefficient of determination (CD) = 0.598, suggesting that the model accounts for approximately 59.8% of the total variance and exhibits strong explanatory power. Residual analysis showed that the means and covariances of the observed variables’ residuals were close to zero, further confirming the adequacy of the model fit. Overall, the fit indices consistently indicate that the constructed model demonstrates good fit quality, effectively capturing the data structure and the relationships among variables). The results are presented in Table 6.

**Table 6:** Moderating effect of secure attachment on the sleeping quality → insomnia index → psychological resilience pathway.

Path	Secure attachment (case 253)	Insecure attachment (case 311)	Intergroup differences (χ^2^ Test)	P score
Sleeping quality→ psychological reliance	−0.02 (0.01)*	−0.02 (0.01)**	0.00	0.96
Insomnia index→ psychological reliance	−0.01 (0.01)	−0.01 (0.01)	0.01	0.91
Sleeping quality→ Insomnia index	1.20 (0.07)***	1.42 (0.07)***	5.10	0.02

***p < 0.001, **p < 0.05, *p < 0.1.

The findings indicate that adult attachment significantly moderated the sleep quality → insomnia pathway (χ^2^(1) = 5.10, *p* = 0.0239), suggesting that the effect of sleep quality on the insomnia index varies across different attachment styles. However, attachment style did not significantly moderate the pathways of insomnia index → psychological resilience or sleep quality → psychological resilience (*p* > 0.90). This means that although attachment style influences the effect of sleep quality on the insomnia index, it does not further affect changes in psychological resilience.

In terms of the moderating effect of attachment style on the sleep quality→ insomnia index pathway, the coefficient for the insecure attachment group (*β* = 1.4245, *p* < 0.001) was higher than that of the secure attachment group (*β* = 1.2039, *p* < 0.001). This suggests that the impact of sleep quality on the insomnia index is stronger among students with insecure attachment, with a moderation effect strength of approximately 18.3%.

## 4 Discussion

Existing research has paid insufficient attention to sleep problems and psychological resilience among college students, as well as to the mechanisms through which sleep affects psychological resilience. College students experience changes in lifestyle and face stress stemming from study and work, which tend to lead to various sleep disorders. This study has confirmed such a conclusion. These findings indicate that sleep issues among college students require serious attention. Unlike working professionals who face heavy work and life pressures or primary and secondary school students who experience sleeping deprivation due to rigid schedules, college students technically have more flexible sleep schedules that allow them to adjust their bedtime and sleep duration. However, factors such as academic pressure and the increasingly competitive job market further affect their sleep habits and quality. Therefore, sleep problems among college students should not be overlooked and require attention from both educational institutions and society.

In recent years there has been a growing interest among psychologists in the concept of resilience, and most importantly in the cultivation of it as both a trait and a learnable skill (Himmel, 2015). Most existing studies treat psychological resilience as either a moderating variable or an independent variable. Few studies have treated psychological resilience as a dependent variable to explore the mechanisms that influence it. Researches calls for the psychological resilience enhancement in college students due to the soared anxiety among students and the lacking of opportunity for students to experience failure and take responsibility for themselves (Gray, 2015). This study, through empirical analysis, points out that overall, college students’ psychological resilience is at a moderate level, with most students scored moderate scale and above. 17.78% of students exhibit high psychological resilience, but 15.21% fall into the low or extremely low psychological resilience categories. College students face numerous pressures, and given the many functions of psychological resilience, this suggests that their level of resilience still needs to be improved.

The impact of sleep index on psychological resilience has been confirmed in many studies, indicating that poorer sleep quality is associated with lower resilience (Arbinaga, 2018). Insomnia, as a severe form of sleep disorder, also has a negative effect on psychological resilience. A systematic review combined 34 studies found that poor sleep quality and insomnia were associated with stress (Gardani et al., 2022). These factors can all affect psychological resilience. They may lead to emotions such as depression, anxiety and influence an individual’s coping strategies in response to their environment, thereby further impacting psychological resilience (Dong et al., 2024). Attachment security may help individuals to filter out worry and anxiety when sleep (Harvey, 2000), attachment insecurity (vs security) is related to psychiatric disorders the objective was to study the attachment style in insomnia (Palagini et al., 2018). The conclusions of previous studies have been confirmed in this paper. Building on that foundation, this study further examines the effects of sleep, insomnia, and secure attachment on psychological resilience. It also tests the mediating role of insomnia in the relationship between sleep and psychological resilience, as well as the moderating role of secure attachment in the relationship between sleep and insomnia. Sleep quality and insomnia index are significantly positively correlated: poorer sleep quality is associated with higher insomnia, which may contribute to lower psychological resilience. Students with insecure attachment tend to have lower psychological resilience compared to those with secure attachment. Insomnia plays a mediating role in the relationship between sleep quality and psychological resilience, that is, sleep quality not only directly affects psychological resilience but also indirectly influences it through insomnia. 39.24% of the effect of sleep quality on psychological resilience occurs via insomnia. Adult attachment style moderates the effect of sleep quality on the insomnia index: students with insecure attachment are more likely to develop insomnia due to poor sleep quality compared to those with secure attachment.

Studies also show that psychological resilience can be bolstered through various means (Kuang et al., 2024). In a recent meta-analysis of resilience-building programs, interventions designed to foster strengths, coping, and protective factors had an overall positive effect in improving resilience and affecting mental health outcomes (Leppin et al., 2014). Study also shows that sleep hygiene and physical activity interventions may affect the cognitive performance, which is good for psychological resilience (Souissi, M. A, et.al, 2025). Studies suggests that raising college students’ awareness of the importance of sleep, improving their sleep habits like doing more physical activities and indicating them to sleep earlier in the night, enhancing sleep quality, reducing insomnia by help students dealing with anxiety, and fostering secure attachment by supplying more social support for them may all contribute to strengthening their psychological resilience, thereby improving their ability to cope with various challenges.

Study limitations and future directions. Despite its contributions, this study has several limitations. First, Sampling Limitations. The study used convenience sampling, with participants primarily from universities in Beijing. Although common method bias tests were conducted, the lack of rigorous scientific sampling introduces certain limitations. Convenience sampling inherently limits both causal interpretation and representativeness of the findings—a dual methodological constraint central to this research. Future research should employ scientific sampling techniques, such as stratified multi-stage sampling, to obtain a more representative sample and analyze variations across universities, academic years, disciplines, and geographic regions. Second, the structural equation model yielded excellent fit indices (CFI, TLI = 1.000; RMSEA = 0.000), this may indicate that the model is good, but there is still some potential issues such as overfitting or misspecification needs to be taken more care. Overfitting can lead to inflated fit indices that do not generalize well to other populations. To address this concern, future studies are encouraged to replicate the model using independent samples or employ cross-validation techniques (e.g., k-fold cross-validation) to assess the stability of parameter estimates across subsets of data. Additionally, expanding the sample size and ensuring greater heterogeneity in participant characteristics would enhance the external validity of the model and provide a more rigorous test of its robustness. Last, further exploration of psychological mechanisms needs to be conducted. This study provides a preliminary analysis of the relationships between sleep quality, secure attachment, and psychological resilience. Future research should explore additional influencing factors, such as psychological stress, to gain a more comprehensive understanding of the mechanisms underlying the effects of sleep quality and attachment on psychological resilience.

## 5 Conclusion

By collecting data from 585 university students in Beijing, China, we found that sleep quality among college students is problematic, with nearly 30% experiencing insomnia. Through linear regression analysis and structural equation modeling, we discovered that sleep quality and psychological resilience are significant positive associated. Students with better sleep quality have higher possibility to have higher levels of resilience. Sleep quality influences psychological resilience both directly and indirectly through insomnia. The higher the insomnia index, the lower the level of psychological resilience among students. Secure attachment plays a moderating role in insomnia, with securely attached students being less likely to experience insomnia compared to those with insecure attachment. Although this study has certain limitations, it offers valuable insights for improving sleep and enhancing psychological resilience among college students.

## Data Availability

The original contributions presented in the study are included in the article/supplementary material, further inquiries can be directed to the corresponding authors.
